# A Review on the Special Radiotherapy Techniques of Colorectal Cancer

**DOI:** 10.3389/fonc.2019.00208

**Published:** 2019-04-02

**Authors:** Shing Yau Tam, Vincent W. C. Wu

**Affiliations:** Department of Health Technology and Informatics, Faculty of Health and Social Sciences, The Hong Kong Polytechnic University, Kowloon, Hong Kong

**Keywords:** special radiotherapy technique, colorectal cancer, local control, survival benefits, personalized radiotherapy

## Abstract

Colorectal cancer is one of the commonest cancers worldwide. Radiotherapy has been established as an indispensable component of treatment. Although conventional radiotherapy provides good local control, radiotherapy treatment side-effects, local recurrence and distant metastasis remain to be the concerns. With the recent technological advancements, various special radiotherapy treatment options have been offered. This review article discusses the recently-developed special radiotherapy treatment modalities for various conditions of colorectal cancer ranging from early stage, locally advanced stage, recurrent, and metastatic diseases. The discussion focuses on the areas of feasibility, local control, and survival benefits of the treatment modalities. This review also provides accounts of the future direction in radiotherapy of colorectal cancer with emphasis on the coming era of personalized radiotherapy.

## Introduction

Colorectal cancer is one of the most common cancers worldwide. It ranks third in men and second in women in 2012 with 746,000 and 614,000 new cases, respectively (1). About two-third of the cases occurred in sigmoid colon or rectum with majority diagnosed as Stage II or above (2), which requires chemoradiotherapy treatment apart from the primary surgical treatment. The current standard of radiotherapy treatment is 3-dimension conformation radiotherapy (3DCRT), which allows target localization and dose analysis of target volume and organs at risk (OARs) via 3D planning and dose volume histograms. The advance in radiotherapy equipment and treatment planning system allows conformation of radiation dose to target structures and limitation of radiation dose to surrounding normal tissues. This leads to improved tumor control by dose escalation while reducing the incidence of acute and late radiation toxicities to bowel. It has been widely used among medium risk, locally advanced, and inoperable rectal cancers for curative or palliative intent. Intensity-modulated radiotherapy (IMRT), volumetric arc therapy (VMAT), helical tomotherapy and proton therapy for colorectal cancer are under development aiming for better conformality index and OAR sparing than 3DCRT (3–9). However, most of the studies were small scale or dosimetric studies. In addition, limitations of these techniques including organ motion, volume variability and dose inhomogeneity lead to the potential concern of underdosing due to rapid drop-off of dose beyond target volumes, therefore more formal prospective trials are required to establish their clinical benefits over 3DCRT (3, 5, 10, 11).

Despite the advance in conventional treatment delivery technique and protocol, radiotherapy treatment side-effects, local recurrence and distant metastasis are the concerns in colorectal cancer which bring about poor quality of life and high mortality to the patients (12, 13). Because of this, specialized radiotherapy techniques have been introduced and aimed to improve treatment efficacy. They can also serve as alternatives to treat patients who could not undergo conventional treatments including surgery. In this review, the recent development of special radiotherapy techniques for various colorectal tumor stages will be introduced (Table 1) along with the future prospective of radiotherapy developments.

**Table 1:** Survival, tumor regression, and/or toxicity outcomes on various special radiotherapy techniques in colorectal cancer.

**Stage**	**Technique**	**Subject number**	**Alone or commitment treatment?**	**Survival/tumor regression figure**	**Side-effects figure**	**References**
Early	Contact therapy	77	Alone	5-year DFS: 74%	N/A	(14)
		11	With EBRT	LC: 91%	N/A	(15)
		124	Alone or combined with interstitial brachytherapy	LC: 83% (T1), 38% (T2) 6-year OS: 62.4%	Acute toxicity: 35.5% Late toxicity: 22%	(16)
		199	Alone or with EBRT	LC: 71%	N/A	(17)
		83	With EBRT	cCR: 63.8%	Grade 1 rectal ulceration: 30% Grade 1 bleeding: 28% Grade 2 bleeding: 6% Grade 3 late toxicity: Not observed	(18)
		200	Alone or with EBRT	cCR: 72%	Grade 1 rectal ulceration: 30% Grade 1 bleeding: 28% Grade 2 bleeding: 10.5% Grade 3 late toxicity: Not observed	(19)
	Endorectal brachytherapy	32	With local excision	LC: 76%, 5-year DFS: 85% 5-year OS: 78%	Grade 2–3 radionecrosis: 4 cases	(20)
Locally advanced	Endorectal brachytherapy (Operable tumors)	100	As neoadjuvant treatment with total mesorectal surgery	5-year LR: 5% 5-year DFS: 65% 5-year OS: 70%	Grade 2 acute proctitis: 99 cases Grade 3 acute proctitis: 1 case	(21)
		48	As boost after EBRT	R0 resection: 98%	Grade 3 toxicity: 6%	(22)
		16	Combined with EBRT	R0 resection: 100% pCR: 44%	N/A	(23)
		34	As neoadjuvant treatment	R0 resection: 83% pCR: 31%	No increase in grade 3–4 toxicity from RT	(24)
		110	As boost with LCCRT	5-year PFS: 52.0% 5-year OS: 63.6% Freedom from locoregional failure: 85.7%	N/A	(25)
	Endorectal brachytherapy (Inoperable tumors)	50	Alone or with EBRT	Median survival: 25 months (Radical intent), 7.2 months (Palliative intent)	N/A	(26)
		38	With EBRT	2-years local PFS: 42% 2-years OS: 63%	Grade 3 late toxicity: 33% Grade 4 late toxicity: 4%	(27)
	IORT	210	As IOERT with pre- or postoperative CRT	5-year LC: 93% 5-year OS: 69%	Grade 3 acute toxicity: 17% Grade 4 acute toxicity: Not observed	(28)
		99	As IOERT with preoperative CRT	LR: 2% 5-year OS: 79%	Grade 3 or 4 toxicity: Not observed	(29)
		30	As HDR-IORT with preoperative EBRT	5-year LC: 56% 5-year OS: 61%	Postoperative morbidity: 46.7%	(30)
		73	As IOERT with preoperative EBRT	5-year LC: 91.8% Median OS: 88 months Median DFS: 80 months	Postoperative complications: 29.6%	(31)
Recurrent tumor	Endorectal brachytherapy	30	As concomitant treatment with surgery	LC: 64%	N/A	(32)
		38	As concomitant treatment with or without surgery and chemotherapy	Median OS: 15 months	N/A	(33)
		9	Alone or with EBRT	8-year OS: 56%	Grade 3 acute toxicity: 33%	(34)
	IORT	74	As HDR-IORT with or without CRT	5-year LC: 39% 5-year DFS: 23% 5-year OS: 23%	Grade 3 toxicity: 32 incidences Grade 4 toxicity: 8 incidences	(35)
		147	As IOERT with or without CRT	5-year LC: 54.1% 5-year DFS: 34.1% 5-year OS: 31.5%	N/A	(36)
		607	As IOERT with pre- or postoperative EBRT	5-year OS: 30% 10-year OS: 16%	Grade 3 or above toxicity: 11%	(37)
		42	As HDR-IORT with preoperative CRT	2-year OS: 47%	N/A	(38)
		49	As IOERT with preoperative EBRT	5-year LC: 35% 5-year DFS: 20% 5-year OS: 27%	N/A	(39)
		42	As IOERT with or without EBRT	3-year OS: 43%	Severe complications: 45%	(40)
		59	As IOERT with EBRT	5-year OS: 30%	Postoperative complications: 52 incidences	(41)
	Salvage EBRT	103	As CRT with or without surgery	5-year OS: 22% (with surgery), 15% (without surgery)	Grade 3 acute toxicity: 22% Grade 4 acute toxicity: 6% Grade 3 late toxicity: 17%	(42)
		32	Alone	5-year LC: 13.1% 5-year OS: 23.2%	N/A	(43)
		22	As CRT	2-year LC: 74.6% 2-year PFS: 45.4% 2-year OS: 82.0%	Grade 3 or 4 acute toxicity: Not observed Late toxicity: 7 incidents	(44)
	SBRT	23	Alone or with EBRT	4-year LC: 74.3% 4-year OS: 24.9%	Grade 3 toxicity: Not observed Grade 4 toxicity: 4.3%	(45)
		18	Alone	3-year LC: 85.9% 3-year OS: 59.3%	Grade 3 or 4 toxicity: 17%	(46)
Metastatic tumor	SBRT	82	With chemotherapy	4-year LC: 75% 4-year OS: 43%	Grade 3 or above toxicity: Not observed	(47)
		50	Alone or with chemotherapy	3-year LC: 70.6% 3-year OS: 64.0%	Grade 3 or above toxicity: Not observed	(48)
	Radioembolization	531	Alone or with chemotherapy	Median OS: 10.6 months	Grade 3 or 4 toxicity: 176 incidences	(49)
		106	Alone or with chemotherapy	Median OS: 6.7 months	Grade 3 or 4 toxicity: 10.4%	(50)
		58	Alone or with chemotherapy	Median OS: 6 months	N/A	(51)

*N/A: Not reported*.

## Early Stage Disease

### Contact Therapy

Contact therapy in rectal cancer has a long history of development since 1960s by Papillon in Lyon, who used a portable x-ray machine of 50 kV (15). It had a source-to-skin distance of 4 cm and delivered a large endocavitary dose of 10–40 Gy per fraction weekly or biweekly to a total dose of 45–100 Gy. Some older studies in the 1980s to 1990s claimed that contact therapy could achieve good disease control and rectal preservation among early stage rectal tumors (52, 53). However, with the discontinuation of contact x-ray machine production and the development of surgical techniques such as minimally invasive surgical approaches, contact therapy has been largely abandoned. Recently, contact therapy has been reconsidered as one of the conservative and curative treatment option for early stage rectal tumor among inoperable patients because of the introduction of new contact radiotherapy machine Phillips RT 50, which does not require general anesthesia and with minimal morbidity. The machine uses a special applicator-proctoscope of 2.4–3 cm wide to deliver X-ray within the rectum at 50 kV and 10–20 Gy/min. The radiation rapidly decreases in depth with about 50% at 5 mm and 10% at 2 cm depth (14).

In clinical application, Christoforidis et al. (14) demonstrated an acceptable prognosis among stage I rectal cancer with contact therapy alone (5-year disease-free survival of 74%) though the prognosis was not as good as radical surgery. While Gérard et al. (15) claimed that 10 out of 11 inoperable patients achieved local control after contact therapy and external beam radiotherapy (EBRT). Another study by Coatmeur et al. (16) found the control rate of 83% for T1 rectal cancer treated with contact therapy, which is deemed as comparable to surgical method. However, the control rate for T2 tumors was not as satisfactory (38%) while the sphincter preservation was possible in 80% cases. The study by Aumock et al. (17) reported a local control rate (LC) of 71% with contact therapy with or without EBRT in 199 patients with early stage rectal tumor and found that the combination with EBRT significantly improved LC (*p* < 0.001).

Since conventional long-course chemoradiotherapy (LCCRT) without operative management could only achieve low level of pathological complete response (pCR) at about 10–30% (18, 54), Sun Myint et al. (18) suggested using contact therapy when suspicious residual disease of 3 cm or less was indicated after LCCRT or EBRT. Eighty-three patients with initial tumor stage cT2 or cT3 were evaluated and clinical complete response (cCR) was recorded in 53 patients after the contact therapy boost. Moreover, low local relapse (13.2%) was achieved and the non-metastatic regrowth could underwent salvage surgery. Toxicity was acceptable with no late gastrointestinal toxicity reported. The group also studied the impact of contact therapy dose escalation on organ preservation (19). Seventy-two percentage (144) patients achieved initial cCR after contact therapy dose escalation with 16 patients developed local relapse after cCR. Thirty-eight of the remaining 56 patients who had residual tumor underwent immediate salvage surgery. Organ preservation was achieved in 62% patients at median follow-up of 2.7 years while 108 of the 136 remained alive patients were colostomy-free.

Although the results of past clinical studies concluded excellent local control, acceptable toxicity and improved EBRT efficacy by contact therapy on early stage tumors, most of the published researches enrolled selective population. For example, Christoforidis et al. (14) only recruited patients with primary, non-metastatic and ultrasonographically staged T1 or T2 rectal adenocarcinoma within 15 cm of the anal verge while those received a boost of EBRT after contact x-ray or had a follow-up period of <6 months were excluded. Whereas, Aumock et al. (17) recruited primary rectal adenocarcinoma patients who received contact therapy with or without EBRT. Therefore, the conclusion of contact therapy in clinical benefits is difficult to be established and more well-structured clinical trials such as the ongoing international trial of Contact Endoscopic Microsurgery (CONTEM) are necessary to confirm the role of contact therapy in the management of early stage colorectal cancer.

### Endorectal Brachytherapy

High-dose-rate (HDR) endorectal brachytherapy belongs to endocavitary radiotherapy, which uses real-time fluoroscopy guidance, can provide excellent dose conformality around the target with steep dose fall-off. This allows dose escalation without jeopardizing the OAR dose. Unlike contact therapy, endorectal brachytherapy utilizes Iridium-192 (Ir-192) in a remote after-loading system. Ir-192 source has about 10 Ci activity and emits gamma radiation up to 1.4 MeV. In addition, the system also uses special single or double-plane rectal implants with the Paris system utilized for dose specification. Therefore, it allows a greater dose penetration and better dose coverage in larger tumors. This technique was initially used for adjuvant or palliative treatment for rectal cancer (55). Recently, several studies have reported that it was suitable to be used as a preoperative or postoperative treatment modality for different stages of rectal cancer (20–27).

For early stage tumors, endorectal brachytherapy is mainly used as adjuvant treatment for patients who have undergone local excision and not suitable for radical surgery due to various issues including poor medical status and old age. Grimard et al. (20) studied 32 cases with T1 or T2 rectal cancer on the long-term outcomes of endorectal brachytherapy after local excision. Both single and double plane implants used 50 Gy prescription. There were 8 cases of local relapse and the 5-year overall survival (OS) was 78% with sphincter preservation in 27 patients. Therefore, this technique can be considered as an alternative to radical surgery for the elderly or poor general condition cases, especially when the tumor is located at the edge of anorectal junction due to the proximity of the sphincter muscle. However, researches on the use of endorectal brachytherapy are still very limited and larger scale trials are needed to establish its role for early stage tumor.

## Locally Advanced Disease

### Endorectal Brachytherapy

While the advantage of endorectal brachytherapy in early stage tumors is still not fully proven, endorectal brachytherapy has attracted more attention on the treatment for locally advanced rectal cancer including both operable and inoperable tumors. For the operable tumors, endorectal brachytherapy can be administered either alone or as a boost after EBRT and its benefit has been studied intensively in several studies. Vuong et al. (21) investigated the radiation toxicity and local recurrence rate on preoperative HDR endorectal brachytherapy delivering 26 Gy in 4 daily fractions on 100 patients with resectable locally advanced rectal tumors. They recorded grade 2 acute proctitis in 99 patients and grade 3 acute proctitis in 1 patient, who subsequently needed blood transfusion. The 5-year local recurrence rate (LR), DFS and OS were 5, 65, and 70% which were more favorable toxicity than those of the EBRT. With regard to the clinical benefits of endorectal brachytherapy boost after EBRT, Jakobsen et al. (22) reported the effect of LCCRT combined with endorectal brachytherapy in 48 T3 rectal tumor cases in terms of histopathologic tumor regression grade and radiation toxicity. Results indicated that all but 1 patient had R0 resection and incidence rate of 6 and 0% in grade 3 and 4 toxicity, respectively. In addition, Sun Myint et al. (23) compared the complete remission and R0 resection rates among patients treated with endorectal brachytherapy combined with EBRT and conventional preoperative LCCRT. All patients (*n* = 16) in the brachytherapy group presented with R0 resection, whereas the rate was 63% in conventional preoperative LCCRT in previously published reports. The pathological complete remission rate (pCR) was 44% in the brachytherapy group, compared to 12% in the conventional LCCRT patients. The same British group investigated the effect of increasing radiation dose by HDR brachytherapy boost among locally advanced rectal cancers (24), which also showed more patients having R0 resection and pathological complete remission than the conventional LCCRT. Moreover, there was no increase in grade 3 or above radiation toxicity and therefore the group advocated the use of HDR brachytherapy boost for treatment improvement. Appelt et al. (25) conducted a randomized trial to investigate the long-term benefit of adding endorectal brachytherapy boost. Two hundred and forty-eight locally advanced rectal cancer cases were randomly assigned to either LCCRT with brachytherapy boost (10 Gy in 2 fractions) or LCCRT only group. However, the results revealed that there were no significant differences in loco-regional control, 5-year OS and progression-free survival (PFS) despite the improved pathologic tumor regression at the time of surgery. Therefore, the definite benefit of preoperative endorectal brachytherapy for locally advanced rectal cancers is yet to be proven.

While for inoperable patients, the objective of endorectal brachytherapy is to use it as a substitution of radical surgery with or without EBRT for radical or palliative intent. Hoskin et al. (26) conducted a retrospective review of 50 cases treated by HDR brachytherapy with or without EBRT for radical intent or brachytherapy alone for palliative intent. Local tumor response was observed in 84% of patients with 14 patients having complete responses. The median survivals for radical and palliative treatments were 25 months and 7.2 months, respectively, demonstrating endorectal brachytherapy as an effective local treatment for inoperable patients. Besides, Rijkmans et al. (27) evaluated the toxicity and efficacy of HDR endorectal brachytherapy combined with EBRT by a prospective phase I study on 38 patients with inoperable rectal cancer. Tumor response was found in 87.9% of patients with 60.6% patients with complete response, while the 2-year OS was 63%. Despite the high overall response rate, high rate of severe late toxicity was also recorded (31.3%). Therefore, endorectal brachytherapy among inoperable patients with locally advanced rectal cancer warrants further toxicity evaluation before implementing as standard of care.

### Intraoperative Radiotherapy (IORT)

Apart from endorectal brachytherapy, intraoperative radiotherapy (IORT) is another special radiotherapy technique aiming to improve treatment outcomes of locally advanced rectal tumors. It delivers radiation precisely to the tumor or tumor bed when the area is exposed during surgery. Minimal exposure of the OARs can be achieved as they are displaced away from the irradiation site and shielded from radiation. Moreover, it also allows dose escalation beyond the capability of EBRT and reirradiation in recurrent tumors when further irradiation by EBRT is not possible. IORT is usually administered as a boost dose with EBRT by means of electron beam (IOERT), HDR brachytherapy (HDR-IORT), or orthovoltage X-ray (56). IOERT allows treatment depth of 1 cm or more with a range of electron energies available and can be delivered in several minutes, while HDR-IORT allows treatment of all surfaces with flexible template in the expense of longer treatment time. Krempien et al. (28) investigated 210 locally advanced rectal cancer patients treated with total mesorectal excision, IOERT by 6–18 MeV electron beams (10–15Gy) and pre- or postoperative chemoradiation (CRT). The long-term results showed that 5-year OS and LC were 69 and 93% respectively. Therefore, IORT boost seems to be feasible and provides favorable local control. For the comparison between with and without IORT boost, Sadahiro et al. (29) found that local recurrence and 5-year OS in the IORT and surgery only group were 2 vs. 16% (*p* = 0.002) and 79 vs. 58% (*p* = 0.02), respectively. Similar benefit on local control was confirmed by another Italian study (57). Hyngstrom et al. (30) also stressed the acceptable postoperative morbidity (46.7%) with excellent long term outcome unrelated to microscopic margin status among 30 IORT treated patients. Nevertheless, Dubois et al. (31) and Masaki et al. (58) challenged the benefits of IORT as the two series discovered no significant differences in OS and LC between IORT and surgery alone. Moreover, a meta-analysis by Wiig et al. (59) on 18 research papers revealed that IORT did not provide significant benefits in LR and OS. Therefore, further clinical studies are necessary to refine the role of IORT boost to the conventional treatment schedule for locally advanced rectal cancers despite the established technical feasibility of this technique.

## Recurrent Tumor

### Endorectal Brachytherapy and IORT

Despite the advancement of treatment modalities for colorectal cancer, 4–8% patients experienced loco-regional recurrence and surgery remains the main salvage treatment (60). Nevertheless, endorectal brachytherapy can be applied either as concomitant treatment with or without conventional treatment or as HDR-IORT. For endorectal brachytherapy as concomitant treatment, Goes et al. (32) conducted the first study with 30 patients of locally recurrent rectal cancer achieving a LC of 64%, which was comparable with IORT. While Kolotas et al. (33) studied the clinical results of brachytherapy as palliative treatment for recurrent rectal cancer in 38 patients. Median post-brachytherapy OS was 15 months while 34 patients achieved pain relief without acute complications, demonstrating endorectal brachytherapy as a feasible option for palliation of recurrent colorectal cancer. Morimoto et al. (34) reported the long-term follow-up results of 9 locally recurrent rectal cancer patients treated by endorectal brachytherapy and reported a 8-year OS of 56% with 3 patients experienced grade 3 acute toxicity. No severe late toxicity was recorded. In general, the past studies have demonstrated that endorectal brachytherapy was a promising concomitant treatment for locally recurrent rectal cancer with good local control and tolerable toxicity.

Similar to IORT for locally advanced rectal cancer, HDR-IORT and IOERT are options for locally recurrent rectal cancers. Both IORT techniques achieved good local control and survival in this patient group (35–40, 61). Alektiar et al. (35) reported the 5-year OS and LR of 23 and 39%, respectively. Moreover, improved local control could be achieved with negative margin of resection (43% negative margin vs. 26% positive margin, *p* = 0.02). Furthermore, the use of IORT with EBRT was a significant predictor of OS (*p* = 0.04). Another study from the same center also found similar results on the satisfactory treatment outcomes of recurrent rectal cancer by HDR-IORT (38). In general, there are relatively more studies conducted on IOERT. Various series reported that the 5-year OS in patients treated by IOERT were ranged from 18.6 to 31.5% (36, 37, 39–41). Mayo Clinic compared the survival statistics in patients receiving IOERT boost and conventional palliative treatment. Significant difference in 3-year OS was found between IORT (43%) and surgery with or without neoadjuvant EBRT (15%, *p* = 0.002) and surgery with adjuvant EBRT (18%, *p* = 0.005) (40). However, the investigation by the Norwegian Radium Hospital demonstrated no significant benefit of IOERT in any R-stage regarding LR or OS (41). An analysis by the same Norwegian group also showed no significant improvement of LR or OS by IORT boost (59). To summarize, the role of IORT is still not certain and requires further investigations in randomized controlled trial settings.

### Salvage EBRT

As R0 resection rate was found to be only 57% for recurrent rectal cancer and salvage surgery may not be feasible on all patients as the quality of life can be much affected (62–64), salvage EBRT can be an alternative to locally recurrent rectal tumors either with or without previous irradiation. Reirradiation treatment has been historically regarded as unacceptable as it is thought to induce severe late radiation toxicity. However, Mohiuddin et al. (42) revealed that the usage of high irradiation doses did not significantly increase the incidence of long-term side-effects while the 5-year OS was 22 and 15% with or without surgery, respectively. For a Japanese series involving salvage EBRT for locally recurrent rectal cancer treated with initial treatment by surgery alone, the 5-year OS and LC were 23.2 and 13.1%, respectively (43). However, a Korean study suggested the reirradiation group demonstrated poorer 2-year PFS and OS than patients without previous irradiation (44). To further improve treatment outcomes, dose-escalated radiotherapy has been introduced recently, this Korean group suggested radiotherapy dose of 70 Gy or higher as it was associated with a higher 2-year PFS (63.5 vs. 20.8%, *p* = 0.014). While the previous Japanese group suggested 75 Gy or above to be the prescribed biological effective dose for desirable outcomes if the OAR doses are within acceptable levels (43). Thus, salvage EBRT remains a possible option for recurrent tumors and is suggested to elicit its clinical benefits through long term follow-up studies especially in terms of survival benefits.

### Stereotactic Body Radiotherapy (SBRT)

Stereotactic body radiotherapy (SBRT) is a recently developed radiotherapy technique for pelvic recurrences of colorectal cancer, particularly for inoperable patients. Patients underwent CT simulation in supine position with vacuum body immobilization system. High resolution CT images (1 mm axial) offer a higher conformality to target volume and avoidance of OARs than conventional EBRT. Gold fiducial markers may be inserted percutaneously to provide real time fiducial tracking. Also, the stereotactic principles in localization allow higher doses per fraction (about 5–16 Gy) to be delivered to tumors due to reduction in mechanical error margin and better OAR sparing (45, 46). Kim et al. (45) reported the survival and toxicity of 23 recurrent rectal cancer cases treated by SBRT using the CyberKnife system. The median dose prescription was 39 Gy in 3 fractions. The 4-year OS and LC was 24.9 and 74.3%, respectively, and only 1 patient developed grade 4 radiation toxicity, which were comparable with other modalities. More recently, Dagoglu et al. (46) conducted a study on 18 patients with recurrent colorectal cancer. Similar to the first study by Kim et al., CyberKnife system was employed and the median prescribed dose was 25 Gy in 5 fractions. The median OS was 43 months and 3 patients developed severe radiation toxicity. Based on these results, SBRT reirradiation is an efficacious technique in local control of pelvic recurrences with comparable survival outcome to IORT though the number of published reports is still inadequate.

## Metastatic tumor

### SBRT

Colorectal cancer commonly presents oligometastasis in the liver and lungs (65). Although surgical resection can provide survival benefit, majority of metastatic patients are inoperable. SBRT is anticipated to provide good local control as reported in many pelvic recurrent patients and it can be administered to both the liver and lungs. Comito et al. (47) studied the safety and efficacy of SBRT in 82 patients with 1–3 unresectable oligometastatic tumors confined to liver or lung. The dose prescription was 48–75 Gy in 3–4 fractions and delivered by RapidArc technique with thermoplastic body mask for immobilization. The series reported 4-year OS and LC to be 43 and 75%, respectively, with the absence of grade 3 or above toxicity. Jung et al. (48) investigated the outcomes of SBRT for 1–3 lung metastatic lesions in 50 colorectal cancer patients treated with 40–60 Gy in 3–4 fractions. The 3-year OS and LC were 64.0 and 70.6%, respectively. Similar to the study by Comito et al. no grade 3 or above pulmonary complications were observed. Hence, SBRT remains to be a viable treatment option for oligometastasis in the liver and lungs though more clinical trials should be conducted in the area.

### Radioembolization

When the metastatic disease is confined to the liver, radioembolization is a treatment option apart from the conventional systemic chemotherapy. Radioembolization employs Yttrium-90 (Y-90)-impregnated resin or glass-based microspheres such as the commercially available TheraSpheres (BTG, Canada) and SIR-Spheres (Sirtex, Australia). Hepatic metastatic tumors obtain primary vascular supply from the hepatic artery rather than the portal vein in normal liver parenchyma and the microvascular density of the hepatic tumors is much greater than the neighboring normal liver parenchyma. Therefore, the microspheres can be entrapped in hepatic tumors when the microspheres are infused through the hepatic artery. Y-90 emits beta radiation with an average energy of 0.94 MeV and a half-life of 64.1 h. The radiation has a range of 2.5 mm in the tissue and such treatment is also known as intra-arterial brachytherapy. Hickey et al. (49) studied the treatment outcomes of 531 colorectal liver metastases patients treated with Y-90 radioembolization and reported a median OS of 10.6 months with common side-effects including abdominal pain, fatigue and nausea. Thirteen percentage patients experienced grade 3–4 hyperbilirubinemia. Further analysis of the survival prediction showed that performance status, absent of extrahepatic metastases, <25% tumor burden, <2 chemotherapy agents and albumin >3 g/dL were the independent factors. These predictive factors were similar to the study by Damm et al. (50) which reported a median OS of 6.7 months in 106 patients. Janowski et al. (51) also obtained similar median OS of 6 months in the study of 58 patients. These recent reports demonstrated that Y-90 radioembolization could lead to promising survival outcomes with acceptable levels of toxicity.

## Discussion

Radiotherapy for colorectal cancer is under intensive development with various special techniques proposed in recent decades for different tumor stages from early stage to metastatic disease. They aimed to treat inoperable patients due to elderly or poor general conditions, patients with advanced stage or recurrent tumors, or acting as a boost of conventional treatment to improve disease control. Despite the published reports demonstrated promising benefits in local control and/or survival with toxicity well-addressed, majority of studies recruited limited and selective populations with various survival, tumor control or toxicity figures reported. Therefore, well-structured phase III clinical trials are warranted to establish these techniques as standard of care.

While the efficacy of radiotherapy for colorectal cancer has been improved with the technological advancements including more accurate radiation delivery, dose escalation to target and lowered OAR doses, the overall survival rates still have rooms for improvement due to metachronous distant metastases. Furthermore, the incidence of colorectal cancer is still increasing worldwide due to present human development levels (66). The major future challenge is the identification of risk factors that influence the radiosensitivity and metastatic power of tumors. This leads to establishment of treatment protocol for individual patients, which is particularly important for the heterogeneous tumor characteristics of stage II and III colorectal cancers.

One of the possible directions is the investigation of individual tumor characteristics which relates with their radiosensitivity and metastatic power. For instance, the concepts of cancer stem cell and tumor hypoxia have been associated with lower radiosensitivity and higher metastatic power (67, 68). Cancer stem cell is a recently developed concept, which is a small population of cancer cells that are regarded as precursors of metastases (69). Whereas, hypoxia has long been considered as an important factor for the failure of radiotherapy and is related to increased radioresistance and higher tendency of metastasize (67, 70). Fractionated treatment could partially solve the issue by tumor reoxygenation, yet short course preoperative radiotherapy (SCPRT) in colorectal cancer may affect the effectiveness of reoxygenation and lead to treatment failure (71). Therefore, these issues should be investigated in the future so as to establish as predictive radiosensitivity and metastatic power biomarkers in the potential development of personalized radiotherapy treatment in future.

## Conclusion

Various special radiotherapy techniques for colorectal cancer have been suggested for different tumor stages. Although the past studies presented encouraging results in disease control and toxicity figures, more phase III clinical trials are needed to verify these techniques as standard of care. Furthermore, individual tumor characteristics such as concepts of cancer stem cell and tumor hypoxia are expected to influence treatment and survival outcome and should be established as a future direction of colorectal cancer radiotherapy development.

## Author Contributions

ST collected information and drafted the manuscript. VW designed the outline and edited the manuscript. All authors read and approved the final manuscript.

### Conflict of Interest Statement

The authors declare that the research was conducted in the absence of any commercial or financial relationships that could be construed as a potential conflict of interest.
